# Associations among maternal prenatal depression, maternal autism traits, and child autism traits in the Environmental Influences on Child Health Outcome (ECHO) program

**DOI:** 10.1186/s11689-026-09709-w

**Published:** 2026-05-21

**Authors:** Chaela Nutor, Anne L. Dunlop, Patricia A. Brennan

**Affiliations:** 1 Department of Psychology, Emory University, 36 Eagle Row, Atlanta, GA 30322 USA; 2 Neil Hodgson Woodruff School of Nursing, Emory University, Atlanta, USA

**Keywords:** Autism spectrum disorder, Maternal depression, Prenatal depression, Maternal autism traits

## Abstract

**Background:**

Maternal depression during pregnancy has been associated with increased risk of offspring autism spectrum disorder (ASD) – a highly heritable neurodevelopmental disorder. However, no study to date has taken into account maternal ASD traits in the association between maternal prenatal depression and child ASD.

**Methods:**

This study utilized data from the Environmental Influences on Child Health Outcomes (ECHO) consortium—a large, national prospective longitudinal study—to examine maternal ASD traits as a covariate and moderator in the association between maternal prenatal depression and child ASD traits. Participants were 645 mother–child dyads. Mothers self-reported prenatal depressive symptoms and ASD traits. Child ASD traits were rated by parents using the Social Responsiveness Scale and Child Behavior Checklist. Maternal depression and child ASD diagnoses were either parent-reported or from medical record review.

**Results:**

We found that both maternal prenatal depressive symptoms and depression diagnoses predicted child ASD traits (β’s > .04, *p*’s < .003). These associations remained significant after accounting for maternal ASD traits for the CBCL only (β’s > .42, *p*’s < .001). Maternal prenatal depression did not predict child ASD diagnoses. Maternal ASD traits predicted child ASD traits and diagnoses (β’s > .06, *p*’s < .001). Maternal ASD traits did not moderate the association between maternal prenatal depression and child ASD traits.

**Conclusion:**

Providers might consider early screening for ASD in children of mothers with a history of elevated depressive symptoms during pregnancy.

**Supplementary Information:**

The online version contains supplementary material available at https://doi.org/10.1186/s11689-026-09709-w.

## Introduction

Autism spectrum disorder (ASD) is a neurodevelopmental disorder characterized by restrictive, repetitive behaviors and atypical social interaction and communication styles [1]. The prevalence and global burden of autism spectrum disorder (ASD) has been increasing over the past several decades [2, 3]. There has been a plethora of research investigating the genetics of and environmental risk factors for ASD. However, the etiology of this disorder is still not well understood which impedes progress on prevention and interventions. Researchers are increasingly investigating perinatal risk factors since the onset of ASD is so early in life. For example, prenatal maternal factors such as low socioeconomic status, stress, and mental illness have been associated with ASD risk [4].

Maternal depression during pregnancy has been consistently associated with a higher risk of children developing ASD. In one study, mothers with a depression diagnosis prior to childbirth had an almost 50% increased risk of having a child with an ASD diagnosis [5]. However, these associations are specific to diagnoses of ASD, which only represents those who had access to receiving these diagnoses and might miss those lacking access to care and those with subthreshold symptoms in the general population. More recently, Avalos et al. [6] found a dose–response association such that more severe maternal depressive symptoms were associated with more ASD traits in the national Environmental Influences on Child Health Outcomes (ECHO) consortium. More research examining how maternal depressive symptomatology might impact child ASD traits is needed.

Notably, most studies linking perinatal risk factors to ASD outcomes do not control for maternal ASD traits. Maternal ASD traits are associated with child ASD traits [7–9] and, more specifically, prenatal maternal ASD traits are associated with later child ASD traits [10]. Thus, an important confound may not be accounted for in existing prenatal maternal depression research focused on ASD.

It is also possible that maternal ASD traits alter the relationship between maternal depression and child ASD. Lin et al. [11] found that maternal ASD traits moderated the association between parenting style and behavior problems of children with ASD. No study to date has evaluated if and how maternal prenatal depression interacts with maternal ASD traits to predict ASD outcomes.

This study aimed to examine the associations among prenatal maternal depression, maternal ASD traits, and child ASD traits in a large, nationwide consortium. We hypothesized that: (1) higher maternal prenatal depressive symptoms and maternal depression diagnoses would both be associated with more child ASD traits and diagnoses; (2) maternal ASD traits would predict child ASD outcomes; (3) maternal prenatal depression would predict child ASD independent of maternal ASD traits; and (4) the association between maternal prenatal depression and child ASD traits would be stronger in cases of higher maternal ASD traits.

## Methods

The present study uses data from the 2nd release of Environmental Influences on Child Health Outcomes (ECHO) program [12, 13]. The ECHO program, established by the National Institutes of Health (NIH), consists of a collection of prospective cohort studies with the purpose of examining the impacts of chemical, biological, physical, and social factors on child health and development [14]. All cohort-specific protocols were approved by the institutional review boards with jurisdiction in each study location, and all participants provided informed consent. De-identified, individual-level datasets of maternal depression and child characteristics were transferred to Emory University under data use agreements. Participants were included if they had data available on mother’s prenatal depressive symptoms, mother’s ASD traits, and at least one of the ASD outcome measures. In cases where more than one child from the same household participated in ECHO, we selected the first enrolled child who had the available data.

### Maternal Prenatal Depression

Maternal depressive symptoms during pregnancy were measured using the Patient-Reported Outcomes Measurement Information System Depression test bank (PROMIS-D; [15]). The PROMIS-D depression item bank consists of 28-items scored on a 5-point Likert scale [16]. Scores from four additional measures including the Edinburgh Postnatal Depression Scale (EPDS, [17] were converted to the PROMIS-D T-score metric and harmonized for use across ECHO (for more information about validation see [18].

Prenatal maternal depression diagnosis were determined via self-report or from medical health records. Medical records were reviewed to determine if the biological mother was diagnosed with depression disorder for the period from 4 weeks prior to the last menstrual period before the pregnancy through 8 weeks postpartum.

### Maternal ASD Traits

The Social Responsiveness Scale adult form measures ASD traits (SRS-2; [19]. Mothers rated their behavior over the past 6 months with a four-point Likert scale (M_age_ of child = 11.76). The SRS has five subscales: social communication, social awareness, social cognition, social motivation, and restrictive interests/repetitive behaviors. The sum of all items was computed to obtain a total score with a higher score indicating more ASD traits. The adult SRS has a specificity level of 0.60 and sensitivity of 0.86 [20].

### Child ASD

The Child Behavior Checklist (CBCL) measures behavioral and emotional problems in children and adolescents [21]. The school-age (age 6 to 18 years) CBCL was used in this study (M_age_ = 11.33). T-scores on the Withdrawn, Social Problems and Thought Problems subscales were summed to create a CBCL ASD-related behavior score for each child. This ASD score has previously been found to discriminate between children with and without ASD, particularly among high-functioning youth [22].

The Social Responsiveness Scale (SRS-2) is commonly used as screening tool for ASD that measures deficits in social behavior. Standardized T-scores from both the full-length 65-item and the 16-item short form SRS were used to assess social impairments during the school-age (4 to 18 years) time period (M_age_ = 10.60; [19]).

ASD diagnoses were determined either via parent-report or medical records. Parents responded to a question asking whether the child had an ASD diagnosis from a physician, psychologist, or healthcare provider. For children from cohorts specifically recruiting individuals at high risk for ASD, ASD status was determined via the Autism Diagnostic Observation Schedule (ADOS-2; [23]).

### Covariates

Child sex was obtained from parent-report or abstracted from medical records. Maternal and paternal age at the time of delivery was determined from demographic questionnaires and medical records. Gestational age at birth was based on prioritization of available reports using the following ordered sources: 1) maternal medical records, 2) childbirth medical records, 3) maternal self-report, or 4) caregiver self-report. For sibling ASD status, parents reported if the enrolled child had any sibling(s) that had a doctor/healthcare provider diagnosis of ASD. Maternal education was the highest level reported during any timepoint during the child’s life. Prenatal tobacco use was determined based on self-reported use of 1) cigarettes, 2) e-cigarettes, and 3) other forms of tobacco (i.e. cigars, hookah, chewing tobacco/snuff, nicotine gum or lozenges, pipes) during pregnancy.

### Data Analyses

**Fig. 1:**
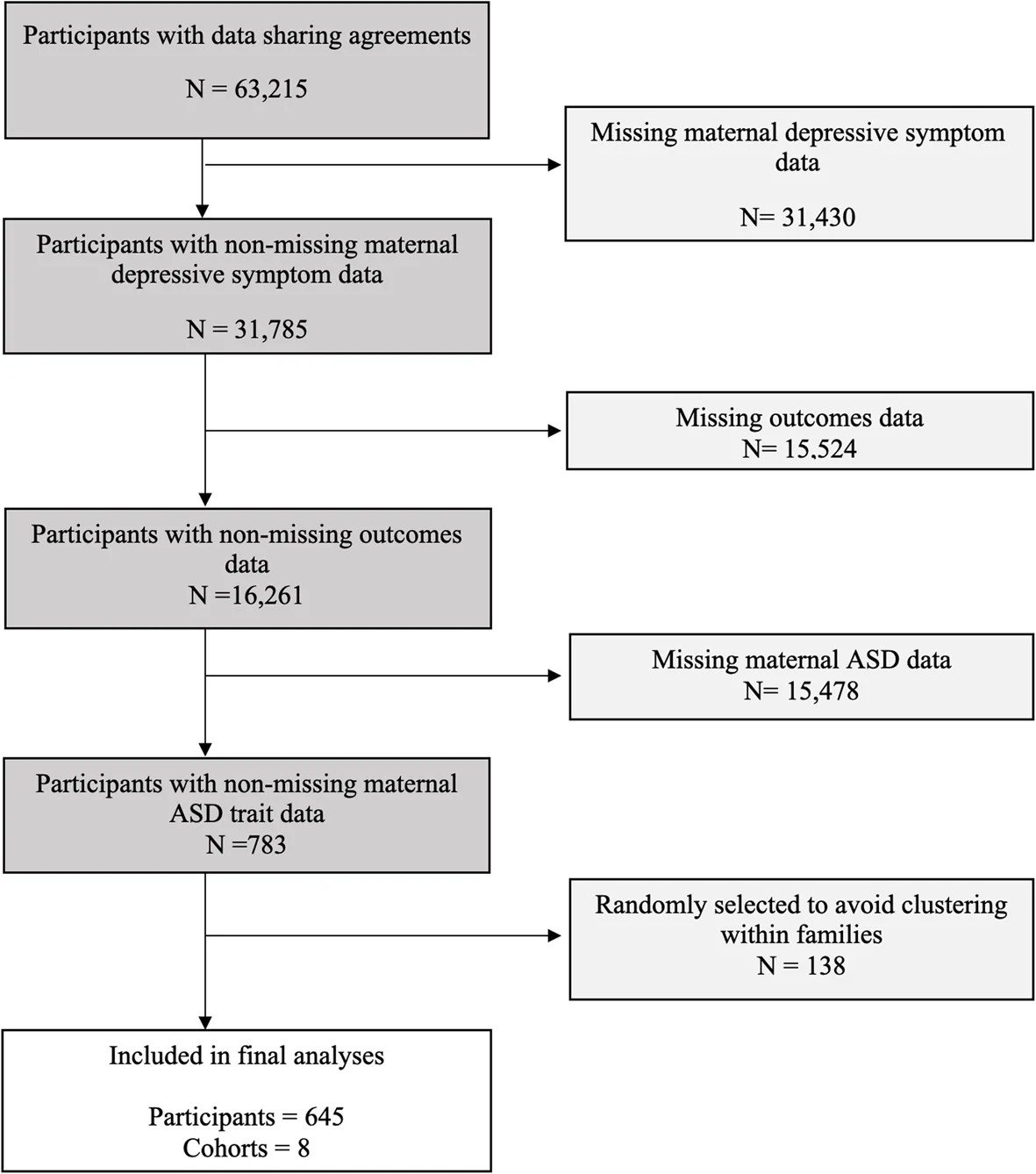
Inclusion in the Analysis

Participants were included in the final dataset if they had maternal prenatal depressive symptom data, maternal ASD trait data, and at least one measure of ASD-related behaviors (*n* = 645; Fig. 1). Missing values were imputed using the Multivariate Imputation by Chained Equations (MICE) package in R [24]. Before imputation, we had 316 valid CBCL and 416 valid SRS,percent missing was 51% and 35.5%, respectively. To assess missingness, Little's Missing Completely at Random (MCAR) test was performed in SPSS using the outcome variables [25]. The test was not statistically significant at the 0.05 level, indicating that the data were likely missing completely at random (x^2^(1) = 5.77, *p* = 0.06).

All analyses utilized generalized linear mixed models (GLMM) with random effects for individual cohorts to account for clustering within cohorts. GLMM was also used to account for covariates. Specifically, we controlled for child sex, gestational age at birth, maternal age, paternal age, maternal education (as a proxy for socioeconomic status), and prenatal tobacco exposure in all analyses. Additionally, we adjusted for age at assessment in our analyses of CBCL scores; however, as SRS scores are age standardized, no age adjustment was necessary for this scale measure. Maternal prenatal depressive symptoms and diagnoses were examined in separate models for all aims. To test Hypothesis 3, we put maternal prenatal depression and maternal ASD traits in the same step of the regression model. To test Hypothesis 4, an interaction variable was used to examine the interaction between maternal ASD traits and maternal prenatal depression. Statistical significance for main aims was determined by a more stringent threshold of *p* < 0.008 for all aims to adjust for multiple comparison using Bonferroni correction. Analyses were conducted in IBM SPSS Version 29.0.2.0.

## Results

Table 1 displays the descriptive characteristics of our entire sample. One hundred and thirteen (18%) mothers had a depression diagnosis. Table 2 displays the descriptive characteristics of our sample divided by maternal prenatal depression status (yes or no). Mothers without depression were more likely to use tobacco during pregnancy than mothers with depression. Otherwise, there were no differences in the demographics of our sample based on predictor variables. Table 3 shows bivariate correlations between covariates and outcome variables. Child biological sex (*r*’s < −0.11, *p*’s < 0.01) and gestational age at birth (*r*’s > 0.14, *p*’s < 0.001) were significantly correlated with all three ASD outcome measures. Prenatal tobacco use was significantly correlated with ASD diagnosis (*r* = 0.10, *p* = 0.02), but not the parent-reported measures. Maternal education was only significantly correlated with the SRS (*r* = 0.10, *p* = 0.01). Maternal age at birth was not correlated with any of the ASD measures. As expected, the parent-reported child ASD measures were significantly correlated with one another (*r* = 0.63, *p* < 0.001). Maternal depressive symptoms and maternal ASD traits were moderately correlated (*r* = 0.26, *p* < 0.001).

**Table 1:** Descriptive Characteristics of Entire Sample

Infant Sex, N(%)	
Male	411 (64)
Child’s Race, N(%)	
White	452 (70)
Black	76 (12)
Asian	18 (3)
Multirace	94 (15)
Other	4 (1)
Child’s Ethnicity, N(%)	
Hispanic	124 (19)
Gestational Age at Birth (completed weeks), M(SD)	35.89 (5.56)
Birth Weight (grams), M(SD)	2,853.28(1,186.92)
Maternal Age at Birth (years), M(SD)	31.55 (5.50)
Paternal Age (years), M(SD)	33.53 (6.22)
Marital Status, N(%)	
Single	136 (21)
Education, N(%)	
High School Diploma or Less	75 (12)
Income, N(%)	
Below the poverty level	108 (17)
Prenatal Tobacco Use, N(%)	50 (8)
CBCL ASD Score, M(SD)	168.57 (20.21)
CBCL Age, M(SD)	11.33 (3.46)
SRS Score, M(SD)	52.43 (3.10)
SRS Age, M(SD)	10.60 (3.67)
Autism Diagnosis, N(%)	188 (29)
Autism Diagnosis Age, M(SD)	4.51 (3.10)

*N* = Number of participants, *% =* Percentage, *M* = Mean, *SD* = Standard deviation, *SRS* = Social Responsiveness Scale, *CBCL* = Child Behavior Checklist

**Table 2:** Descriptive Characteristics of Sample Divided by Prenatal Depression

Total Sample *N* = 645	Depressed Mothers *N* = 113	Non-Depressed Mothers *N* = 532	Significance (*p*-values)
Infant Sex, N(%)			.05
Male	63 (10)	348 (54)	
Child’s Race, N(%)			.48
White	83 (13)	369 (57)	
Black	9 (1)	67 (10)	
Asian	1 (0)	17 (3)	
Multirace	19 (3)	75 (12)	
Other	1 (0)	4 (1)	
Child’s Ethnicity, N(%)			.74
Hispanic	23 (4)	101 (16)	
Gestational Age at Birth (completed weeks), M(SD)	36.37 (5.18)	35.79 (5.64)	.31
Birth Weight (grams), M(SD)	2,898.03 (1,048.89)	2,843.78 (1,214.91)	.66
Maternal Age at Birth (years), M(SD)	31.09 (5.84)	31.65 (5.42)	.32
Marital Status, N(%)			.83
Single	23 (4)	113 (18)	
Education, N(%)			.36
High School Diploma or Less	16 (3)	59 (9)	
Income, N(%)			.26
Below the Poverty Level	23 (4)	85 (13)	
Prenatal Tobacco Use, N(%)	15 (2)	35 (5)	.02*

*N* = Number of participants, *% =* Percentage, *M* = Mean, *SD =* Standard deviation

^*^Difference between depressed mothers and non-depressed mothers is significant at the 0.05 level using Pearson Chi-Square (2-tailed)

**Table 3:** Bivariate Correlations Between Covariates and Outcomes

Covariates		Outcomes		
		School-Age SRS	School-Age CBCL	Autism Diagnosis
Child Biological Sex	Pearson Correlation	-.121^**^	-.105^**^	-.229^***^
	Sig. (2-tailed)	.002	.008	<.001
Maternal Age at Birth	Pearson Correlation	.005	-.029	-.076
	Sig. (2-tailed)	.897	.458	.054
Gestational Age at Birth	Pearson Correlation	.223^**^	.274^***^	.144^***^
	Sig. (2-tailed)	<.001	<.001	<.001
Sibling ASD Status	Pearson Correlation	-.02	-.02	.01
	Sig. (2-tailed)	.60	.64	.88
Maternal Education	Pearson Correlation	.102^*^	.060	.009
	Sig. (2-tailed)	.010	.128	.816
Prenatal Tobacco	Pearson Correlation	.018	-.010	.095^*^
	Sig. (2-tailed)	.646	.808	.016

*SRS =* Social Responsiveness Scale, *CBCL* = Child Behavior Checklist

^*^Correlation is significant at the 0.05 level (2-tailed)

^**^Correlation is significant at the 0.01 level (2-tailed)

^***^Correlation is significant at the 0.001 level (2-tailed)

All primary results are shown in Table 4. The full models including covariate contributions can be found in Supplemental Materials. We found that maternal prenatal depressive symptoms predicted child ASD traits measured by school-age SRS scores (β = 0.04, F = 9.26, t = 3.04, *p* = 0.002), and CBCL ASD scores (β = 0.43, F = 36.47, t = 6.04, *p* < 0.001), but not ASD diagnoses (β = 01, F = 2.33, t = 1.53, *p* = 0.13; Supplemental Table 1). Similarly, we found that maternal depression diagnoses predicted ASD traits measured by school-age SRS scores (β = 0.98, F = 10.09, t = 3.18, *p* = 0.002), and CBCL (β = 11.11, F = 33.77, t = 5.81, *p* < 0.001), but not ASD diagnoses (β = 0.06, F = 2.19, t = 1.48, *p* = 0.14; Supplemental Table 2).

**Table 4:** Linear Regression Models Examining Main Effect of Prenatal Maternal Depressive Symptoms on Child ASD Traits

	SRS			CBCL			ASD Diagnosis				
	Est.	t	Sig.	Est.	t	Sig.	Est.	t		Sig.	
Hypothesis 1											
Prenatal Maternal Depressive Symptoms	.04	3.01	.003^*^	.50	6.01	<.001^**^	.003	1.38		.17	
Prenatal Maternal Depression	.98	3.19	.002^*^	11.13	5.81	<.001^**^	.07	1.54		.12	
Hypothesis 2											
Maternal ASD Traits	.06	4.86	<.001^**^	.42	5.42	<.001^**^	27.77	5.27		<.001^**^	
Hypothesis 3a											
Maternal ASD Traits	.05	4.29	<.001^**^	.33	4.22	<.001^**^	.009	5.08		<.001^**^	
Prenatal Maternal Depressive Symptoms	.03	2.01	.05	.42	4.95	<.001^**^	<.001	.24		.81	
Hypothesis 3b											
Maternal ASD Traits	.05	4.35	<.001^**^	.35	4.51	<.001^**^	.01	5.06		<.001^**^	
Prenatal Maternal Depression	.73	2.36	.02	9.54	4.97	<.001^**^	.03	.61		.54	
Hypothesis 4											
Maternal ASD Traits X Maternal Depressive Symptoms	-.001	-.75	.45	.001	.14	.89	<.001	-.07		.95	
Maternal ASD Traits X Depression Diagnosis	.01	.39	.69	.18	1.01	.31	.01	1.79		.07	

Covariates included child biological sex, maternal age, paternal age, gestational age at birth, education, prenatal tobacco use. For CBCL only, age at assessment was included as a covariate

*Est.* = Estimate, *SRS =* Social Responsiveness Scale, *CBCL* = Child Behavior Checklist, *ASD* = Autism spectrum disorder

^*^significant at the 0.008 level (2-tailed)

^**^significant at the 0.001 level (2-tailed)

**Fig. 2:**
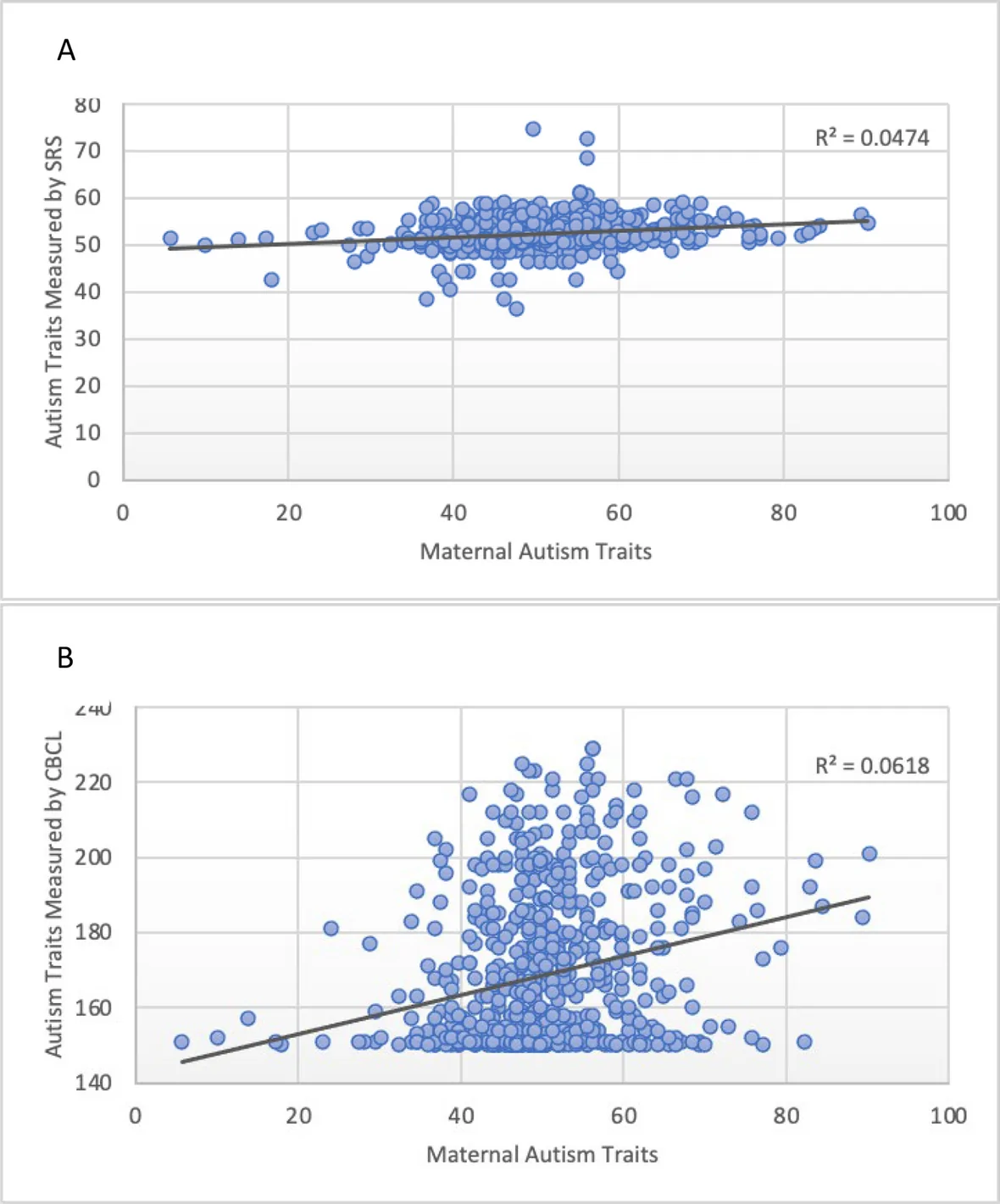
Associations Between Maternal Autism Traits and Child Autism Traits *Note.* Panel **A** depicts the association between maternal ASD traits (x-axis) and child ASD traits measured by the SRS (y-axis). Panel **B** depicts the association between maternal ASD traits (x-axis) and child ASD traits measured by the CBCL (y-axis). In panel B, the y-axis does not start at 0. Numbers reflect raw scores

As hypothesized, maternal ASD traits predicted child ASD traits measured by the SRS (β = 0.06, F = 23.81, t = 4.88, *p* < 0.001) and CBCL (β = 0.43, F = 29.65, t = 5.45, *p* < 0.001; Fig. 2) in addition to ASD diagnosis (β = 0.01, F = 28.85, t = 5.37, *p* < 0.001; Supplemental Table 3).

We partially confirmed our hypothesis that maternal prenatal depressive symptoms would predict child ASD above and beyond maternal ASD traits. When both maternal prenatal depressive symptoms and maternal ASD traits were in the model, maternal prenatal depressive symptoms remained significant predictors for CBCL ASD scores, but not SRS or ASD diagnoses (Supplemental Table 4). Similarly, maternal depression diagnoses also predicted child ASD traits measured by the CBCL above the contribution of maternal ASD traits, but this was not true for the SRS or child ASD diagnoses (Supplemental Table 5).

Contrary to our hypothesis, maternal ASD traits did not significantly moderate the association between maternal prenatal depressive symptoms and child ASD traits (all *p*’s > 0.42). Maternal ASD traits also did not moderate the association between maternal depression diagnoses and child ASD traits or diagnoses (all *p*’s > 0.07).

### Sensitivity Analyses

Analyses were re-run excluding one mother with an ASD diagnosis on record. Analyses additionally excluding 6 fathers with ASD were also conducted. Results remained the same as above in both instances.

## Discussion

Findings from this study show that maternal depression during pregnancy is associated with child ASD traits, but not child ASD diagnoses. Maternal ASD traits were associated with both child ASD traits and diagnoses. When maternal ASD traits were included as a covariate in the model examining the relationship between maternal depression and child ASD, maternal prenatal depressive symptoms remained significant predictors for child ASD traits measured by the CBCL, but not the SRS, or ASD diagnoses. We did not find support for maternal ASD traits as a moderator in the association between prenatal depression and child ASD.

This replicates and adds to previous literature examining the association between maternal prenatal depression and child ASD. Avalos et al. [6] also found that both prenatal depression diagnostic status and prenatal maternal depressive symptoms predicted child ASD traits in the ECHO consortium. Despite the association between maternal prenatal depression and child ASD *traits*, in this study neither maternal prenatal depression symptoms nor diagnoses were associated with child ASD *diagnoses*.

Because ECHO includes some ASD-enriched cohorts, the prevalence of ASD diagnoses in our sample was higher than the general population. Specifically, 29% of our sample had ASD diagnoses from a healthcare provider while the national prevalence is 3% [2]. However, these numbers were lower according to parent reported measures: 14% of our sample were above the clinical cutoff on the CBCL autism scale. These vastly different severity estimations might represent differences between reports from parents and diagnosing providers.When determining diagnostic status, providers use more information than the questionnaires used in this study – which might explain discrepancies. The ASD trait measures may also reflect social functioning deficits more broadly, which suggests that maternal psychopathology might associate with more broad based outcomes rather than ASD per se.

Maternal depression is often treated with therapy or selective serotonin reuptake inhibitors (SSRIs). By and large SSRI use during pregnancy has not been associated with child ASD [26–28]. One study specifically examined this using the ECHO sample and found that the association between antidepressants and child ASD did not withstand controlling for confounders [29]. This suggests that it is the presence of depressive symptoms themselves, not the medications used to treat them, that is contributing to offspring ASD.

Future work should explore mechanisms through which prenatal depression leads to increased ASD traits. Research suggests that inflammation might be implicated. Prenatal depressive symptoms are associated with increased inflammation in infants and inflammation partially mediates the association between prenatal depressive symptoms and early language skills [30]. There is also evidence that inflammation is indicated in the etiology of ASD [31]. For example, elevated levels of maternal inflammatory cytokines during pregnancy are associated with increased risk of ASD in offspring [32]. Cortisol has also been proposed as a mediator between prenatal depression/stress and negative child outcomes [33–35]. Prenatal depression is associated with increased cortisol levels [36]. Davis et al. [37] found that higher prenatal maternal cortisol is associated with better cognitive functioning in offspring. Ram et al. [38] found that fetal exposure to lower levels of maternal cortisol was associated with higher levels of ASD symptoms, but only among male children – which is consistent with the higher prevalence of ASD among males.

We also replicated previous findings that maternal traits of ASD predict child ASD traits, which aligns with robust literature regarding the heritability of both ASD and ASD traits [39, 40]. However, maternal ASD traits did not moderate the association between prenatal depression and child ASD which means that this association did not depend on maternal ASD traits. It is possible that maternal ASD traits predict depression. Hosozawa et al. [41], found that ASD traits during pregnancy predicted postpartum depression. Future studies might examine depression as a mediator in the association between maternal ASD and child ASD.

### Strengths and Limitations

One strength of this study is the examination of the role of maternal ASD traits in the relationship between prenatal depression and child ASD traits in multiple ways: as a predictor, a covariate, and a moderator. In doing so, we thoroughly evaluated the contributions of maternal ASD traits. Another strength of this study is that it leverages data from a large cohort which allowed us to comprehensively characterize our sample by including multiple relevant confounds and assessing ASD with multiple continuous measures. We were able to utilize both dimensional and diagnostic measures of maternal prenatal depression and child ASD to leverage statistical power. Although ECHO is a national cohort, our sample represents only the subset of individuals who had complete data for the analyses, and therefore our results may not generalize across the United States. 

In this study, autism diagnoses were assessed using parent report or medical record review. It is possible that parent reports were biased, especially given that reports of child behavior provided by depressed individuals are more likely to be distorted [42, 43]. Medical record review might be subject to missingness, incomplete records, and clinician bias [44]. Due to high missingness, we were unable to assess paternal ASD traits in addition to maternal ASD traits. Multiple studies have found that paternal ASD traits predicted child ASD traits [45, 46]. Alternatively, one study found that paternal ASD traits did not predict child ASD traits using the SRS [47]. Therefore, more research on the association between paternal ASD traits and child ASD traits is needed. Notably, maternal ASD traits do not reflect genetic risk for ASD, so future work could use polygenic risk scores or microRNAs to better address the role of genetics in the association between prenatal depression and child ASD. It is possible that maternal depression alters gene expression that leads to more ASD traits. For example, a recent study found that the interaction between prenatal maternal stress and the *SERT* gene predicts child ASD [48].

Another limitation of this study is that we were unable to examine prenatal timing of maternal depression. Given that fetal neurodevelopment occurs in stages, it has been hypothesized that the timing of exposures may affect risk, with the fetus being more sensitive during some timepoints in pregnancy in comparison to others. For example, Davis et al. [49] found that cortisol levels, a correlate of depression, predicted infant temperament in late pregnancy, but not early pregnancy. More recently, Nutor et al. [50], similarly found that maternal prenatal depressive symptoms measured in late pregnancy, but not early pregnancy predicted ASD traits on the CBCL. Others have found that the timing of exposure to prenatal stress and trauma have different downstream effects as well [51, 52]. It may be that maternal depression does interact with maternal ASD traits during specific developmental windows, but we were unable to assess this possibility with the data available in ECHO.

In conclusion, this study provides evidence that maternal prenatal depression and maternal ASD traits are independently associated with child ASD traits. Furthermore, prenatal depression predicts child ASD traits even when maternal ASD traits are accounted for. Maternal ASD traits did not moderate the association between prenatal depression and child ASD traits. It might be helpful for providers to consider these findings when caring for children of mothers with elevated depressive symptoms during pregnancy, even in cases where there is no family history of ASD.

## Supplementary Information


Supplementary Material 1.


## Data Availability

The datasets supporting the conclusions of this article are available in the Data and Specimen Hub repository, [https://doi.org/10.57982/bg8s-9535].

## Abbreviation

ASD: Autism spectrum disorder
