# Supplementary material for: Associations among maternal prenatal depression, maternal autism traits, and child autism traits in the Environmental Influences on Child Health Outcome (ECHO) program
Source: J Neurodev Disord. 2026 May 21;18:50. doi: 10.1186/s11689-026-09709-w (PMC13536670; doi:10.1186/s11689-026-09709-w)
Supplement: Supplementary file 1 [file 11689_2026_9709_MOESM1_ESM.docx]

**Supplemental Table 1.**

*Linear Regression Models Examining Main Effect of Prenatal Maternal Depressive Symptoms on Child ASD Traits*

|  | SRS | | | CBCL | | | ASD Diagnosis | | |
| --- | --- | --- | --- | --- | --- | --- | --- | --- | --- |
|  | Est. | t | Sig. | Est. | t | Sig. | Est. | t | Sig. |
| **Block 1:** Covariates | | | | | | | | | |
| Child Biological Sex | -.43 | -1.71 | .09 | -1.46 | -.94 | .35 | -.15 | -4.16 | <.001^**^ |
| Maternal Age | -.04 | -1.13 | .26 | -.26 | -1.28 | .20 | -.004 | -.94 | .35 |
| Paternal Age | .02 | .57 | .57 | .02 | .13 | .90 | -.004 | -.97 | .33 |
| Gestational Age at Birth | .04 | .96 | .34 | .54 | 2.16 | .03 | <.001 | .00 | 1.00 |
| Sibling ASD Status | .26 | .71 | .48 | 1.18 | .49 | .63 | .14 | 2.60 | .01 |
| Education | .74 | 1.96 | .05 | 2.56 | 1.09 | .27 | -.02 | -.43 | .67 |
| Prenatal Tobacco Use | .19 | .39 | .70 | .04 | .01 | .99 | .21 | 3.13 | .002^*^ |
| Age at Assessment  (CBCL only) |  |  |  | -.63 | -2.69 | .01 |  |  |  |
| **Block 2:** Main Effects | | | | | | | | | |
| Prenatal Maternal Depressive Symptoms | .04 | 3.01 | .003^*^ | .50 | 6.01 | <.001^**^ | .003 | 1.38 | .17 |

*significant at the 0.005 level (2-tailed)

**significant at the 0.001 level (2-tailed)

*Note.* Est. = estimate, SRS = Social Responsiveness Scale, CBCL = Child Behavior Checklist

**Supplemental Table 2.**

*Linear Regression Models Examining Main Effect of Prenatal Maternal Depression Diagnosis on Child ASD Traits*

|  | SRS | | | CBCL | | | ASD Diagnosis | | |
| --- | --- | --- | --- | --- | --- | --- | --- | --- | --- |
|  | Est. | t | Sig. | Est. | t | Sig. | Est. | t | Sig. |
| **Block 1:** Covariates | | | | | | | | | |
| Child Biological Sex | -.45 | -1.80 | .07 | -1.65 | -1.06 | .29 | -.15 | -4.21 | <.001^**^ |
| Maternal Age | -.03 | -1.06 | .29 | -.23 | -1.11 | .27 | -.004 | -.91 | .36 |
| Paternal Age | .02 | .54 | .59 | .02 | .11 | .91 | -.004 | -.98 | .33 |
| Gestational Age at Birth | .05 | 1.23 | .23 | .68 | 2.71 | .01 | .001 | .12 | .91 |
| Sibling ASD Status | .34 | .92 | .36 | 1.83 | .75 | .45 | .15 | 2.71 | .01 |
| Education | .73 | 1.94 | .05 | 2.66 | 1.13 | .26 | -.02 | -.42 | .67 |
| Prenatal Tobacco Use | .09 | .18 | .85 | -1.39 | -.46 | .65 | .21 | 2.99 | .003^*^ |
| Age at Assessment  (CBCL only) |  |  |  | -.79 | -3.39 | <.001^**^ |  |  |  |
| **Block 2:** Main Effects | | | | | | | | | |
| Prenatal Maternal Depression | .98 | 3.19 | .002^*^ | 11.13 | 5.81 | <.001^**^ | .07 | 1.54 | .12 |

*significant at the 0.005 level (2-tailed)

** significant at the 0.001 level (2-tailed)

*Note.* Est. = estimate, SRS = Social Responsiveness Scale, CBCL = Child Behavior Checklist

**Supplemental Table 3.**

*Linear Regression Models Examining Maternal ASD Traits as Main Effects of Child ASD Traits*

|  | SRS | | | CBCL | | | ASD Diagnosis | | |
| --- | --- | --- | --- | --- | --- | --- | --- | --- | --- |
|  | Est. | t | Sig. | Est. | t | Sig. | Est. | t | Sig. |
| **Block 1:** Covariates | | | | | | | | | |
| Child Biological Sex | -.39 | -1.56 | .12 | -.87 | -.55 | .58 | 18.08 | -4.25 | <.001^**^ |
| Maternal Age | -.03 | -.86 | .39 | -.18 | -.88 | .38 | .46 | -.68 | .50 |
| Paternal Age | .02 | .65 | .51 | .04 | .23 | .82 | .76 | -.87 | .38 |
| Gestational Age at Birth | .04 | 1.17 | .25 | .67 | 2.65 | .01 | .01 | .11 | .91 |
| Sibling ASD Status | .24 | .66 | .51 | .98 | .40 | .69 | 6.46 | 2.54 | .01 |
| Education | .77 | 2.08 | .04 | 2.88 | 1.22 | .22 | .05 | -.23 | .82 |
| Prenatal Tobacco Use | .12 | .25 | .80 | -.62 | -.20 | .84 | 8.64 | 2.94 | .003 |
| Age at Assessment  (CBCL only) |  |  |  | 12.96 | -3.60 | <.001^**^ |  |  |  |
| **Block 2:** Main Effects | | | | | | | | | |
| Maternal ASD Traits | .06 | 4.86 | <.001^**^ | .42 | 5.42 | <.001^**^ | 27.77 | 5.27 | <.001^**^ |

*significant at the 0.005 level (2-tailed)

**significant at the 0.001 level (2-tailed)

*Note.* Est. = estimate, SRS = Social Responsiveness Scale, CBCL = Child Behavior Checklist

**Supplemental Table 4.**

*Linear Regression Models Examining Prenatal Maternal Depressive Symptoms and Maternal ASD Traits as Main Effects of Child ASD Traits*

|  | SRS | | | | CBCL | | | ASD Diagnosis | | |
| --- | --- | --- | --- | --- | --- | --- | --- | --- | --- | --- |
|  | Est. | t | | Sig. | Est. | t | Sig. | Est. | t | Sig. |
| **Block 1:** Covariates | | | | | | | | | | |
| Child Biological Sex | -.43 | | -1.72 | .09 | -1.45 | -.94 | .35 | -.147 | -4.25 | <.001^**^ |
| Maternal Age | -.03 | | -.91 | .36 | -.21 | -1.05 | .30 | -.003 | -.68 | .50 |
| Paternal Age | .02 | | .67 | .50 | .04 | .25 | .80 | -.003 | -.87 | .38 |
| Gestational Age at Birth | .04 | | 1.03 | .31 | .57 | 2.30 | .02 | .001 | .09 | .92 |
| Sibling ASD Status | .21 | | .58 | .56 | .76 | .32 | .75 | .133 | 2.53 | .01 |
| Education | .79 | | 2.14 | .03 | 3.01 | 1.30 | .19 | -.012 | -.22 | .82 |
| Prenatal Tobacco Use | .11 | | .23 | .82 | -.52 | -.18 | .86 | .197 | 2.94 | .003 |
| Age at Assessment  (CBCL only) |  | |  |  | -.71 | -3.09 | .002^*^ |  |  |  |
| **Block 2:** Main Effects | | | | | | | | | | |
| Maternal ASD Traits | .05 | | 4.29 | <.001^**^ | .33 | 4.22 | <.001^**^ | .009 | 5.08 | <.001^**^ |
| Prenatal Maternal Depressive Symptoms | .03 | | 2.01 | .05 | .42 | 4.95 | <.001^**^ | <.001 | .24 | .81 |

*significant at the 0.01 level (2-tailed)

**significant at the 0.001 level (2-tailed)

*Note.* Est. = estimate, SRS = Social Responsiveness Scale, CBCL = Child Behavior Checklist

**Supplemental Table 5.**

*Linear Regression Models Examining Prenatal Maternal Depression Diagnosis and Maternal ASD Traits as Main Effects* *of Child ASD Traits*

|  | SRS | | | CBCL | ASD Diagnosis | | | | | |
| --- | --- | --- | --- | --- | --- | --- | --- | --- | --- | --- |
|  | Est. | t | Sig. | Est. | | t | Sig. | Est. | t | Sig. |
| **Block 1:** Covariates | | | | | | | | | | |
| Child Biological Sex | -.45 | -1.80 | .07 | -1.63 | | -1.06 | .29 | -.15 | -4.29 | <.001^**^ |
| Maternal Age | -.03 | -.86 | .39 | -.18 | | -.89 | .37 | -.003 | -.68 | .50 |
| Paternal Age | .02 | .66 | .51 | .04 | | .24 | .81 | -.003 | -.87 | .38 |
| Gestational Age at Birth | .04 | 1.21 | .23 | .68 | | 2.77 | .01 | .001 | .12 | .91 |
| Sibling ASD Status | .26 | .72 | .47 | 1.27 | | .53 | .60 | .13 | 2.56 | .01 |
| Education | .79 | 2.13 | .03 | 3.13 | | 1.35 | .18 | -.01 | -.22 | .83 |
| Prenatal Tobacco Use | .03 | .07 | .95 | -1.79 | | -.60 | .55 | .19 | 2.88 | .004^*^ |
| Age at Assessment  (CBCL only) |  |  |  | -.85 | | -3.71 | <.001^**^ |  |  |  |
| **Block 2:** Main Effects | | | | | | | | | | |
| Maternal ASD Traits | .05 | 4.35 | <.001^**^ | .35 | | 4.51 | <.001^**^ | .01 | 5.06 | <.001^**^ |
| Prenatal Maternal Depression | .73 | 2.36 | .02 | 9.54 | | 4.97 | <.001^**^ | .03 | .61 | .54 |

*significant at the 0.005 level (2-tailed)

**significant at the 0.001 level (2-tailed)

*Note.* Est. = estimate, SRS = Social Responsiveness Scale, CBCL = Child Behavior Checklist
